# The role of capybaras (*Hydrochoerus hydrochaeris*) as hosts for hemoplasmas in an urban park of Brazilian Midwest

**DOI:** 10.1007/s11259-026-11603-8

**Published:** 2026-11-07

**Authors:** Julia Gindri Bragato Pistori, Gabriel Carvalho de Macedo, Heitor Miraglia Herrera, William Oliveira de Assis, Leonardo França do Nascimento, Oscar Fernandes Junior, Filipe Martins Santos, Nayara Yoshie Sano, João Vitor dos Santos da Silva, Victória Valente Califre de Mello, Eliz Oliveira Franco, Ana Cláudia Calchi, Marcos Rogério André, Carina Elisei De Oliveira

**Affiliations:** 1 Parasitic Biology Laboratory, Department of Environmental Sciences and Agricultural Sustainability, Dom Bosco Catholic University, Campo Grande, MS Brazil; 2 Trypanosomatid Biology Laboratory, Department of Parasitic Biology, Oswaldo Cruz Foundation, Rio de Janeiro, RJ Brazil; 3 Department of Biotechnology, Dom Bosco Catholic University, Campo Grande, MS Brazil; 4 Institute of Biosciences, Federal University of Mato Grosso do Sul, Campo Grande, MS Brazil; 5 Facultad de Ciencias Médicas, Universidad del Pacífico, Pedro Juan Caballero, Paraguay; 6 Department of Local Development, Dom Bosco Catholic University, Campo Grande, MS Brazil; 7 Department of Microbiology, Immunology and Parasitology, Rio de Janeiro State University, Rio de Janeiro, RJ Brazil; 8 Vector-Borne Bioagents Laboratory, Department of Pathology, Reproduction and One Health, School of Agricultural and Veterinarian Sciences, São Paulo State University, Jaboticabal, SP Brazil; 9 Departament of Animal Biology, Biology Institute, State University of Campinas, Campinas, SP Brazil

**Keywords:** Rodent, Hemotropic mycoplasma, Hemoparasite, Urban wildlife

## Abstract

Hemoplasmas (hemotropic mycoplasmas) are Gram-negative bacteria that bind to the surface of red blood cells of mammals and may cause hematological alterations in the infected host, and in urban environments, wildlife species may act as important reservoirs of these agents. The study aimed to evaluate, through molecular analysis, the infection of hemoplasmas in capybaras from the Parque das Nações Indígenas, Campo Grande, Mato Grosso do Sul, Brazil. Additionally, we evaluated the health status of infected and non-infected capybaras by haematological parameters. The results of PCR assays based on the 16 S rRNA gene confirmed the presence of hemoplasmas in 84.6% (55/65) of the samples. Phylogenetic inferences based on 16 S and 23 S rRNA genes positioned the sequences into the *´Candidatus* Mycoplasma haematohydrochoerus*´* clade. The findings suggest that the high prevalence, low genetic variability, and unchanged hematological parameters may indicate host tolerance or adaptation to *‘Ca*. M. haematohydrochoerus’ infection.

**Supplementary Information:**

The online version contains supplementary material available at https://doi.org/10.1007/s11259-026-11603-8.

## Introduction

The capybara (*Hydrochoerus hydrochaeris*) is one of the most common wild mammals found in rural and urban environments across Brazil. It is the largest living rodent on the planet, reaching up to around 100 kg, and exhibit social and hierarchical behaviors, being found in groups of more than 50 individuals including females, offsprings, a dominant male, and subordinate males (Herrera et al. 2011; Macdonald 1981). In urban areas these animals are commonly seen in parks with dense vegetation and bodies of water, generally adapting to human presence and coexisting peacefully with other animal species (Farikoski et al. 2019; Rucco et al. 2020). However, the presence of these rodents in urban centers becomes a problematic situation due to their high infestation by ectoparasites, particularly ticks, which are main vectors of pathogens (Vieira et al. 2009). This scenario may lead to spillover of parasites, with capybaras being source of infection to other animal species and even humans (Ellwanger et al. 2021; Kmetiuk et al. 2025).

Historically, free-living animals are reported infected by different pathogens, being classified as reservoir hosts (Gonçalves et al. 2020; de Mello et al. 2024; Massini et al. 2019), however, there is a gap in knowledge regarding the effects of parasitic infections on the health of wild hosts, even though is known that parasites can induce significant hematological alterations and impact the health and survival of its hosts. In this context, hematological analyses are important tools to assess physiological and metabolic changes resulting from parasitism in wild mammals (Gioia-Di Chiacchio et al. 2014; Nantes et al. 2019; Santos et al. 2018; de Macedo et al. 2026).

Among the hemoparasites found in domestic and wild mammal species, hemoplasmas (hemotropic mycoplasmas), Gram-negative bacteria that bind to the surface of red blood cells in mammals (Felder et al. 2011; dos Santos et al. 2014), are commonly reported (Vieira et al. 2009; André et al. 2011, 2020; de Sousa et al. 2017; de Mello et al. 2019, 2024; Souza et al. 2019; Gonçalves et al. 2020; Mongruel et al. 2022; Santana et al. 2022; Oliveira et al. 2023; Perles et al. 2023). During the infection, the severity of clinical signs varies according to factors such as hemoplasma species, bacteria load, host health status, and co-infections (dos Santos et al. 2014; Kmetiuk et al. 2025). Although some cases may present severe hemolytic anemia, accompanied by lethargy, weight loss, and weakness (dos Santos et al. 2014; Tagawa et al. 2012), infected animals are often asymptomatic. In addition, transmission routes differ among hemoplasma species and may include direct blood contact during aggressive interactions, transplacental transmission, or transmission via hematophagous arthropod vectors (Museux et al. 2009; de Souza Ferreira et al. 2024; Millán et al. 2024).

Recently, a new species of hemoplasma was detected in capybaras from anthropogenic areas of Brazil (Vieira et al. 2021; Gonçalves et al. 2020). In this sense, the present study aimed to investigate the occurrence and the genetic variability of hemoplasmas in a population of capybaras that live in a large urban park in the Brazilian Midwest, as well as to evaluate the influence of hemoplasma infection in hematological parameters.

## Materials and methods

### Capture, collection of biological samples, and population structure of capybaras

This study was conducted in the Parque das Nações Indígenas (PNI), an urban park located in Campo Grande municipality, Mato Grosso do Sul state, Brazilian Midwest (20°27′22.5”S, 54°34′52.5”W) (Online Resource 1). From March 2023 to June 2024, capybaras were captured using CO₂-powered darts containing ketamine (10 mg/kg) and xylazine (0.5 mg/kg), with doses based on visually estimated body weight. After sedation, animals were weighed, aged and sexed, and had physiological parameters monitored, followed by blood collection from the femoral vein and identification with subcutaneous transponders. Additionally, population observations were conducted to assess group structure and size through a single observer, which conducted weekly monitoring sessions over the course of a year. The observations began at 4 pm, a time when the animals begin to move from the forested area to the open areas of the park, allowing for better visualization and recording of the individuals. All the field procedures were conducted in accordance with the Instituto Chico Mendes de Conservação da Biodiversidade (ICMBio) (license number 89588-1), the Instituto de Meio Ambiente de Mato Grosso do Sul (IMASUL) (license number 83/048580/2023) and Ethics Committee for Animal Use (CEUA) of Universidade Católica Dom Bosco, Campo Grande, MS (009/2023).

### DNA extraction and quality assessment

Blood samples were subjected to DNA extraction using the DNeasy Blood & Tissue Kit (Qiagen^®^, United States) according to manufacturer’s instructions. The extracted DNA was quantified through spectrophotometry (Biodrop^®^, United States), and the DNA viability was attested by a conventional Polymerase Chain Reaction (cPCR) targeting a 227 bp fragment of the mammalian interphotoreceptor retinoid-binding protein (*irbp*) gene using the primers IRBP-CF-FWD (5’-TCCAACACCACCACTGAGATCTGGAC-3’) and IRBP-CF-REV (5’-GTGAGGAAGAAATCGGACTGGCC-3’), as described by Ferreira et al. (2010).

### Molecular detection of hemoplasmas

For hemoplasma DNA detection, we performed screening cPCR targeting the 16 S rRNA gene using two pairs of primers (1° round: Myco16S-322s and Myco16S-938as - fragment of ∼620 bp; 2° round: HemMycop16S-41s and Myco16S-938as - fragment of ∼900 bp) as described by Maggi et al. (2013). Then, two positive samples from each capybara group were submitted to a PCR targeting the 23 S rRNA gene using the primers 23S_HAEMO_F and 23S_HAEMO_R (fragment of ∼800 bp) (Mongruel et al. 2020). The positive controls consisted of DNA of bat blood positive for *Mycoplasma* sp. (de Mello et al. 2024), and sterile negative control consisted of ultrapure water (Invitrogen^®^, United States). The PCR products were subjected to horizontal electrophoresis in 1% agarose gel stained with Ethidium Bromide (0.5 µL/ML), using a 100 bp molecular weight marker (Life Technologies^®^, United States). The results were visualized in an ultraviolet light transilluminator coupled to analysis software (ChemiDoc MP Imaging System, Bio-Rad^®^, United States).

Twelve PCR products from each one of the screening and characterization assays were purified using the Wizard SV Gel and PCR Clean-Up System kit (Promega^®^, United States), according to the manufacturer’s recommendations, and the genomic material was quantified using NanoDrop (Thermo Scientific^®^, United States). The purified amplicons were submitted to Sanger sequencing in ABI PRISM 3700 platform (Applied Biosystems^®^, United States) at the Center for Biological Resources and Genomic Biology (CREBIO) of the Department of Technology of the College of Agrarian and Veterinary Sciences (FCAV, UNESP, Jaboticabal, São Paulo, Brazil).

### BLASTn, phylogenetic and genetic diversity analyses

The BLAST program was used to compare the obtained sequences with those previously deposited in GenBank (www.ncbi.nlm.nih.gov/genbank). The sequences were aligned with other sequences of the same gene using the MAFFT software, version 7 (Katoh et al. 2019). Maximum likelihood (ML) phylogenetic analysis was inferred on the IQ-TREE online server (Nguyen et al. 2015) using 1000 replicates of UFBoot2 – Ultrafast Bootstrap (Hoang et al. 2018). The evolutionary model was found using the jModelTest2 software, based on the Akaike Information Criterion (AIC), via the CIPRES portal (Miller et al. 2010). Editing of phylogenetic trees as well as outgroup rooting was performed using Treegraph 2.0.56–381 beta software (Stover et al. 2010). Genetic diversity analysis was performed using sequences obtained from the 16 S rRNA and 23 S rRNA gene using the DnaSP v6 software (Rozas et al. 2017).

### Hematological analysis

Red blood cells (RBC) and white blood cells (WBC) were measured in Neubauer chambers, hematocrit (Hct) was obtained using a microhematocrit centrifuge (Labolan^®^, Brazil), and hemoglobin (Hb) was measured in a biochemical analyzer (Bioplus^®^, Brazil). The mean corpuscular volume (MCV), the mean corpuscular hemoglobin concentration (MCHC) and mean corpuscular hemoglobin (MCH) were calculated based on RBC, Hb and Hct values.

### Statistical analysis

The associations of hemoplasma occurrence according to sex, age, and among groups were assessed using the Chi-square test with a 95% confidence interval. For each hematological parameter, descriptive statistics (mean ± standard deviation) were calculated according to sex and age. Then we compared the means of each variable according to sex an age using Student’s T and Mann-Whitney U tests according to data distribution, which was accessed through a Shapiro-Wilk test. Variables were considered statistically significant for p values < 0.05. All data were analyzed using R software (R Core Team 2023).

## Results

### Populational structure and capture of capybaras

It was possible to count 220 individuals inhabiting the PNI, which are divided into six segregated groups, ranging from 17 to 61 individuals per group. Of the estimated 220 individuals within the park, 65 were captured, comprising 38 females (22 adults and 16 juveniles) and 27 males (15 adults and 12 juveniles).

### Molecular detection of hemoplasmas

All capybara blood DNA samples were positive in the PCR targeting the *irbp* gene. From the 65 blood samples tested, 55 (84.6%) were positive for hemoplasmas in the screening PCR. Positivity for hemoplasmas was observed in all six capybara groups in the PNI, with the number of positive individuals per group ranging from 2 to 18, and in one of the groups, all captured animals (*n* = 18) tested positive (Table 1). No significant difference was observed in the occurrence of hemoplasma according to sex (*p* = 0.8092), age (*p* = 0.128), and groups (*p* = 0.3184).

**Table 1:** Fieldwork and molecular results of capybaras from Parque das Nações Indígenas, Campo Grande, Mato Grosso do Sul, Brazil

Group	Estimated capybaras	Captured capybaras	Female 33/38 (86.8%)		Male 22/27 (81.4%)		Molecular positivity for hemoplasmas
			Adult	Young	Adult	Young	
G1	17	3/17 (17.6%)	1/1	0/0	1/1	0/1	2/3 (66.6%)
G2	28	5/28 (17.8%)	1/1	1/2	2/2	0/0	4/5 (80%)
G3	54	16/54 (29.6%)	4/5	2/4	3/3	4/4	13/16 (81.2%)
G4	25	13/25 (52%)	7/7	0/1	4/5	0/0	11/13 (84.6%)
G5	61	18/61 (29.5%)	5/5	8/8	2/2	3/3	18/18 (100%)
G6	35	10/35 (28.5%)	3/3	1/1	1/2	2/4	7/10 (70%)
Total	220	65/220 (29.5%)	21/22	12/16	13/15	9/12	55/65 (84.6%)

Positive for hemoplasmas/captured

### Sequence analysis

BLASTn analysis of the 16 S rRNA gene revealed high similarity (≈ 99.87–100%) between the samples and *Mycoplasma* sp. previously identified in capybaras from Foz do Iguaçu and Campo Grande, Brazil. Similarly, the 23 S rRNA gene sequences showed high identity (99.88–100%) with *Mycoplasma* sp. reported in *H. hydrochaeris* from Rio Grande do Sul and Foz do Iguaçu. All sequences generated in this study were deposited in GenBank under accession numbers PV607972–PV607983 (16 S rRNA) and PV625064–PV625075 (23 S rRNA), and detailed information is provided in the Online Resource 2.

**Fig. 1:**
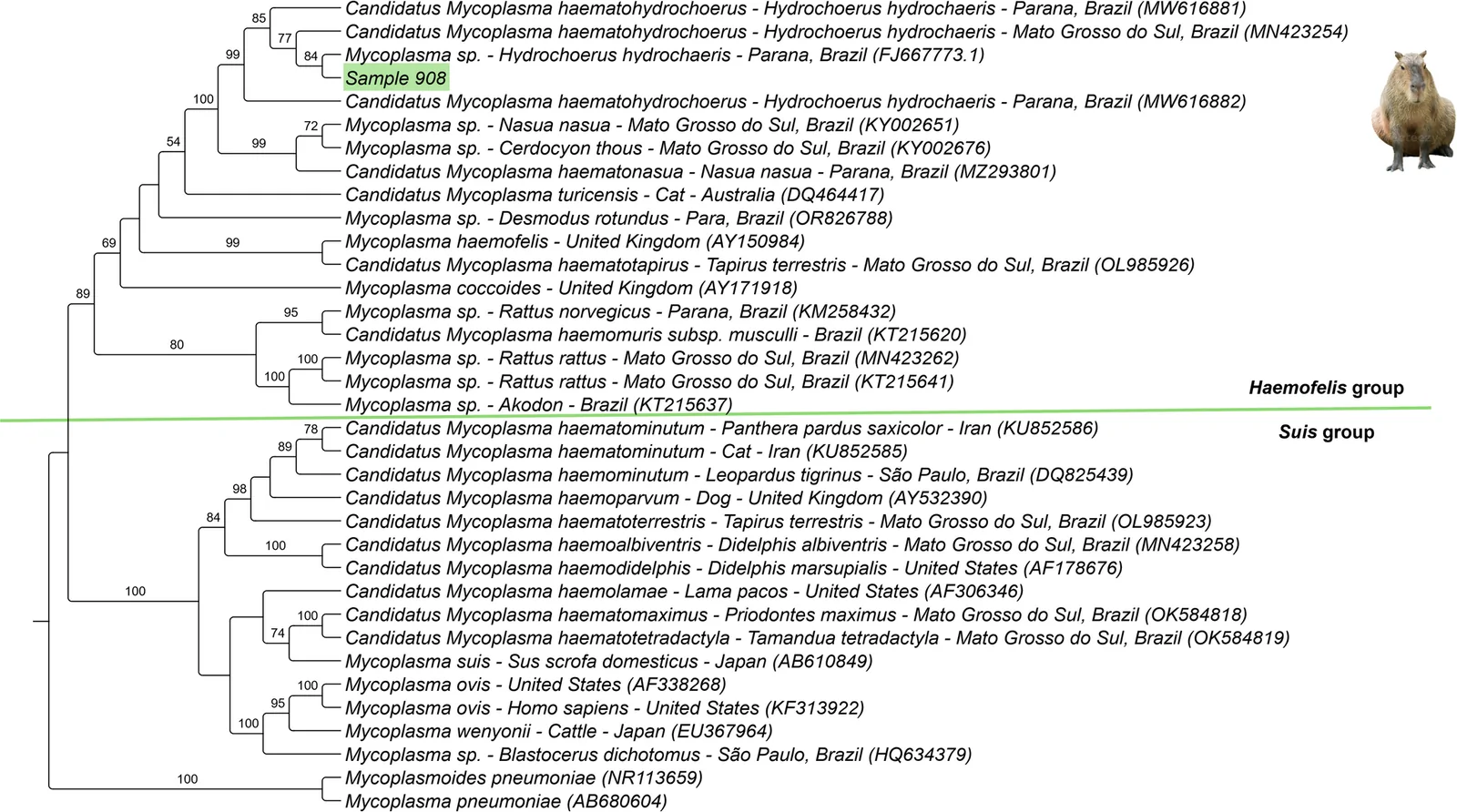
Phylogenetic tree based on 900 bp alignment of the 16 S rRNA gene of *Mycoplasma* spp., inferred by the Maximum Likelihood method and GTR + I + G evolutionary model. *Mycoplasmoides pneumoniae* and *Mycoplasma pneumoniae* were used as an outgroup

**Fig. 2:**
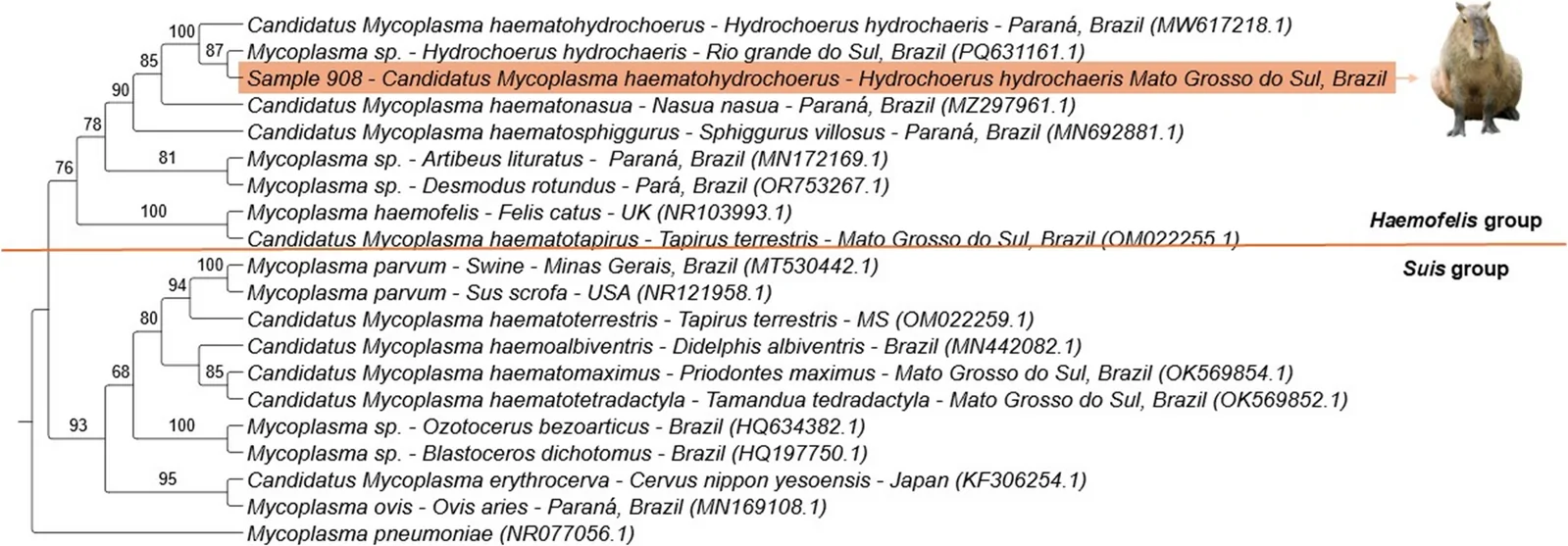
Phylogenetic tree based on 800 bp alignment of the 23 S rRNA gene of *Mycoplasma* spp., inferred by the Maximum Likelihood method and TIM2 + I + G evolutionary model. *Mycoplasma pneumoniae* was used as an outgroup

For the 16 S rRNA and 23 S rRNA genes, the best-fit evolutionary models were GTR + I + G and TIM2 + I + G, respectively. Phylogenetic analyses were conducted using 12 sequences obtained in this study, together with 34 (16 S rRNA) and 19 (23 S rRNA) sequences retrieved from GenBank. Based on analyses performed using the DnaSP software, all 12 sequences identified herein belonged to the same haplotype [haplotype 1] for both genes, therefore, a single representative sequence (#908) was selected for phylogenetic reconstruction to improve tree clarity. All sequences obtained in this study clustered within the *haemofelis group*, and the 16 S rRNA-based phylogenetic tree showed that the representative hemoplasma sequence clustered within the same clade as ‘*Candidatus* Mycoplasma haematohydrochoerus’, with high bootstrap support (Fig. 1), while the 23 S rRNA-based phylogenetic tree placed this sequence within the same clade as sequences previously detected in capybaras from Rio Grande do Sul, Brazil (PQ631161.1), and ‘*Ca*. M. haematohydrochoerus’ identified in a capybara from Paraná, Brazil (MW617218) (Fig. 2).

### Hematological analysis results

Hematological analyses were performed on 57 samples due to the requirement for immediate processing of fresh blood samples. No significant results were observed among the recorded averages of the hematological values of 47 animals positive and 10 animals negative for hemoplasmas. The results regarding the mean and standard deviation values are shown in the Online Resource 3.

## Discussion

The detection of hemoplasmas in capybaras in Brazil was initially reported by Vieira et al. (2009), in a study carried out in the state of Paraná, in which 64% (20/31) of the animals tested positive by PCR for *Mycoplasma coccoides*. However, analysis of the 16 S rRNA gene from the positive samples revealed low identity with *M. coccoides* and with *M. haemomuris*, possibly indicating a new species. Later, Vieira et al. (2021) confirmed this hypothesis and identified this novel agent as ‘*Ca.* M. haematohydrochoerus’. In this work, we confirmed the occurrence of ‘*Ca.* M. haematohydrochoerus’ in capybaras in the state of Mato Grosso do Sul, with an occurrence rate of 84.6% (55/65), higher than the occurrence previously reported by Gonçalves et al. (2020) in three urban forest fragments of Campo Grande, including the PNI [50% (7/14)]. The sample size recorded here (nearly one third of estimated capybaras in PNI) is quite high compared to other studies with free-ranging capybaras, and provided a better representation of the whole capybara population, improving the statistical robustness of the results and allowing more reliable conclusions on the frequency of hemoplasma infection and its genetic evaluation.

Although the life cycle of ‘*Ca.* M. haematohydrochoerus’ is not yet fully known, previous studies have detected hemoplasma DNA in ticks collected from infected animals, supporting the hypothesis of tick-mediated transmission (Gonçalves et al. 2020). In this context, the presence of tick infestation observed in capybaras by Gonçalves et al. (2020) and in the present study (JGBP, personal communication), suggests the participation of ticks on transmission cycle of ‘*Ca.* M. haematohydrochoerus’. This risk is particularly relevant in areas with dense vegetation, preferred locations for ticks (Dias et al. 2020 and JGBP, personal communication), likely facilitating the transmission and maintenance of hemoplasmas. This association is reinforced by the 100% occurrence of hemoplasmas in the G5 group, whose capybaras inhabit a dense forest area (Online Resource 1).

The absence of a significant difference in the prevalence of hemoplasmas between young and adult capybaras observed in the present study, with high infection rates in both age groups, may indicate early infection. Although, transplacental transmission remains a speculative route and requires further studies, Millán et al. (2024) suggest that this route is relevant in the epidemiology of hemoplasmas. Additionally, transmission of hemoplasmas through aggressive interactions was experimentally reported in other mammal species (Museux et al. 2009) and may contribute to the maintenance and spread of the agent, as capybaras exhibit territorial and hierarchical behaviors (Herrera and Macdonald 1993).

Analysis of the phylogenetic tree based on the 16 S rRNA gene reveals a clear evolutionary distance between the hemotropic *Mycoplasma* detected in capybaras and those associated with other rodents, such as *Rattus* spp. and *Akodon* spp. (Fig. 1). Although they belong to the haemofelis group, the different genotypes identified in rodents show divergences in ancestry between species. This phylogenetic differentiation is consistent with the former taxonomic separation between the hosts, as capybaras belong to the infraorder Hystricomorpha, and rats and related species are included in Myomorpha. The evolutionary divergence between these rodent lineages may have favored the co-specialization of hemotropic mycoplasmas, resulting in a genetically distinct grouping in capybara parasites (Vieira et al. 2009, 2021). Moreover, previous phylogenetic studies in other mammal species highlight host specificity among hemplasma species (Collere et al. 2021; Mongruel et al. 2022).

Haplotype analysis based on 16 S rRNA sequences revealed the presence of a single haplotype (Hap_1) in all capybara groups, resulting in zero haplotype diversity and indicating low genetic variability of hemoplasmas within the PNI. Furthermore, haplotype network analysis demonstrated that all previously reported 16 S rRNA sequences for capybaras in Brazil also cluster within the same haplotype (Hap_1), reinforcing the absence of detectable genetic structuring between different geographic regions of Brazil (Midwest and South). A similar pattern was observed for the 23 S rRNA gene, for which all analyzed sequences corresponded to a single genetic variant, further corroborating the circulation of a genetically homogeneous lineage in Brazilian capybaras.

We found no significant differences in the hematological variables between parasitized and non-parasitized animals. Moreover, the sampled capybara population appeared to be in good physical condition, reproduced adequately (indicated by the constant presence of many young animals in the groups), and the average values of RBC, Hb and Hct found in the positive capybaras are in accordance with the reference values published in AZA (2021). Taken together, these findings suggest that capybaras living in the PNI exhibit a potential tolerance or adaptation to ‘*Ca*. M. haematohydrochoerus’ infection. However, it has been reported that adhesion of *Mycoplasma* to the erythrocyte surface can induce structural membrane alterations, leading to increased immune recognition and erythrocyte clearance, ultimately resulting in hemolytic anemia (Messick. 2004; Vieira et al. 2009, 2021; Rosanna et al. 2020). In this sense, it is necessary to monitor the hematological status of the capybara population of the PNI over time in order to prevent any clinical condition related to anemia.

The molecular data revealed low genetic variability for the 16 S and 23 S rRNA genes, corroborating the maintenance of a genetically homogeneous lineage of ‘*Ca*. M. haematohydrochoerus’ in the PNI. Because this organism has been recently described, further research is needed to clarify its transmission dynamics, host range, pathogenic potential, and ecological factors influencing its spread. In fact, our study was limited by the lack of molecular screening of salivary glands of ticks associated with capybaras, as well as specific analyses to validate transplacental transmission and transmission through aggressive interactions, possibly through experimental analyses. The results presented here, along with those reported by Vieira et al. (2009; 2021), reinforce the hypothesis that ‘*Ca.* M. haematohydrochoerus’ circulates widely across different Brazilian ecotypes, establishing the capybara as a major reservoir. Given the large human presence and public access in the PNI, continuous surveillance is essential to monitor this pathogen’s dynamics and ensure both human and animal health.

## Supplementary Information

Below is the link to the electronic supplementary material.

Supplementary Material 1 (PDF 260 KB)

Supplementary Material 2 (PDF 168 KB)

Supplementary Material 3 (PDF 125 KB)

## Data Availability

The datasets presented in this study can be found in online repositories. The names of the repository/repositories and accession number(s) can be found in the article/Supplementary Material.
