# Supplementary material for: The role of capybaras (Hydrochoerus hydrochaeris) as hosts for hemoplasmas in an urban park of Brazilian Midwest
Source: Vet Res Commun. 2026 Nov 7;51(1):42. doi: 10.1007/s11259-026-11603-8 (PMC13744522; doi:10.1007/s11259-026-11603-8)
Supplement: Supplementary file 1 [file 11259_2026_11603_MOESM1_ESM.pdf]

Figure S1 Study area: Parque das Nações Indígenas in Campo Grande, Mato Grosso do Sul, Brazil, showing the spatial distribution of capybaras population in six groups (G1 – G6).

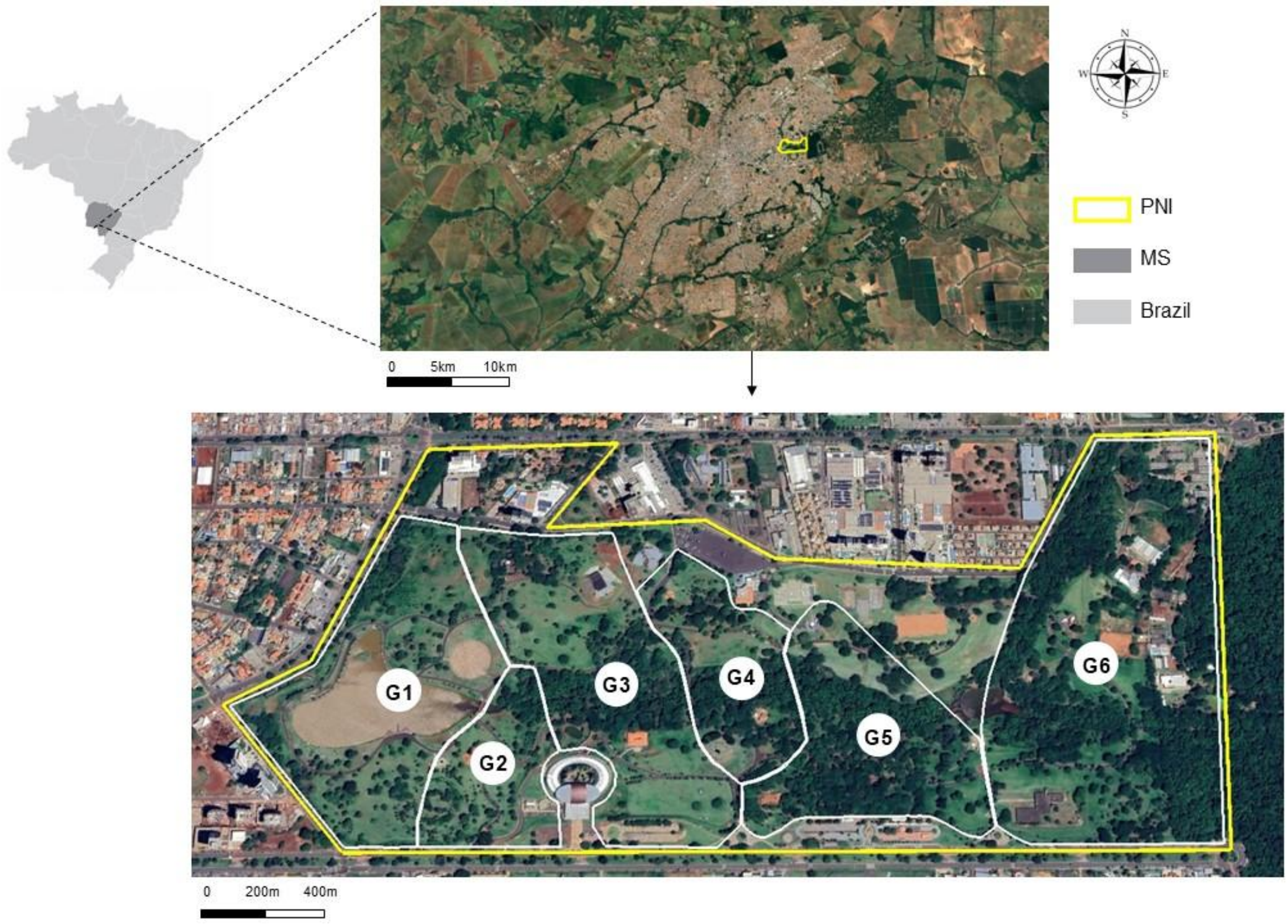

The role of capybaras (*Hydrochoerus hydrochaeris*) as hosts for hemoplasmas in an urban park of Brazilian Midwest

Veterinary Research Communications

Julia Gindri Bragato Pistori[1]; Gabriel Carvalho de Macedo[1,2]; Heitor Miraglia Herrera[1,3]; William Oliveira de Assis[1]; Leonardo França do Nascimento[4]; Oscar Fernandes Júnior[5]; Filipe Martins Santos[1,3,6,7] Nayara Yoshie Sano[1]; João Vitor dos Santos Alves da Silva[8]; Victória Valente Califre de Mello[8]; Eliz Oliveira Franco[8]; Ana Cláudia Calchi[8,9]; Marcos Rogério André[8]; Carina Elisei De Oliveira[1,3]

[1]Parasitic Biology Laboratory, Department of Environmental Sciences and Agricultural Sustainability, Dom Bosco Catholic University, Campo Grande, MS, Brazil

[2]Trypanosomatid Biology Laboratory, Department of Parasitic Biology, Oswaldo Cruz Foundation, Rio de Janeiro, RJ, Brazil

[3]Department of Biotechnology, Dom Bosco Catholic University, Campo Grande, MS, Brazil

[4]Institute of Biosciences, Federal University of Mato Grosso do Sul, Campo Grande, MS, Brazil.

[5]Facultad de Ciencias Médicas, Universidad del Pacífico, Pedro Juan Caballero, Paraguay.

[6]Department of Local Development, Dom Bosco Catholic University, Campo Grande, MS, Brazil.

[7]Department of Microbiology, Immunology and Parasitology, Rio de Janeiro State University, Rio de Janeiro, RJ, Brazil.
[8]Vector-Borne Bioagents Laboratory, Department of Pathology, Reproduction and One Health, School of Agricultural and Veterinarian Sciences, São Paulo State University, Jaboticabal, SP, Brazil.
[9]Departament of Animal Biology, Biology Institute, State University of Campinas, Campinas, SP, Brazil.

Corresponding author e-mail: juliapistori1@gmail.com (Julia Gindri Bragato Pistori)
