# Supplementary material for: The role of capybaras (Hydrochoerus hydrochaeris) as hosts for hemoplasmas in an urban park of Brazilian Midwest
Source: Vet Res Commun. 2026 Nov 7;51(1):42. doi: 10.1007/s11259-026-11603-8 (PMC13744522; doi:10.1007/s11259-026-11603-8)
Supplement: Supplementary file 2 [file 11259_2026_11603_MOESM2_ESM.pdf]

Table S1 BLASTn results of the 16S and 23SrRNA sequences detected in blood samples of capybaras (*Hydrochoerus hydrochaeris*) captured in an urban park of Campo Grande, Mato Grosso do Sul, Brazil.

Animal code	Social group	Size (bp)	rRNA	Genbank accession number	Query cover	Identity	Genbank homologous sequence
UCDB 914	G1	812	16S	PV607974	100%	100%	FJ667773.1
UCDB 958		816	16S	PV607982.2	100%	100,00%	FJ667773.1
UCDB 915	G2	760	16S	PV607975	100%	100%	FJ667773.1
UCDB 936		808	16S	PV607978	100%	99.87%	FJ667773.1
UCDB 908	G3	768	16S	PV607972	100%	100%	FJ667773.1
UCDB 935		817	16S	PV607977	100%	100%	FJ667773.1
UCDB 942	G4	636	16S	PV607979	100%	100%	MN423255
UCDB 963		665	16S	PV607983	100%	100%	MN423255
UCDB 910	G5	769	16S	PV607973	100%	100%	FJ667773.1
UCDB 948		815	16S	PV607980	100%	100%	FJ667773.1
UCDB 926	G6	792	16S	PV607976	100%	99.87%	FJ667773.1
UCDB 954		814	16S	PV607981	100%	100%	FJ667773.1
UCDB 914	G1	734	23S	PV625066	100%	100%	PQ631161.1
UCDB 958		770	23S	PV625074	100%	100%	PQ631161.1
UCDB 915	G2	788	23S	PV625067	100%	100%	PQ631161.1
UCDB 936		786	23S	PV625070	100%	100%	MW617211
UCDB 908	G3	768	23S	PV625064	100%	100%	PQ631161.1
UCDB 935		772	23S	PV625069	100%	100%	PQ631161.1
UCDB 942	G4	692	23S	PV625071	100%	100%	PQ631161.1
UCDB 963		776	23S	PV625075	100%	100%	PQ631161.1
UCDB 910	G5	768	23S	PV625065	100%	100%	PQ631161.1
UCDB 948		780	23S	PV625072	100%	100%	PQ631161.1
UCDB 926	G6	777	23S	PV625068	100%	100%	PQ631161.1
UCDB 954		815	23S	PV625073	100%	99,88%	PQ631161.1

The role of capybaras (*Hydrochoerus hydrochaeris*) as hosts for hemoplasmas in an urban park of Brazilian Midwest

Veterinary Research Communications

Julia Gindri Bragato Pistori[1]; Gabriel Carvalho de Macedo[1,2]; Heitor Miraglia Herrera[1,3]; William Oliveira de Assis[1]; Leonardo França do Nascimento[4]; Oscar Fernandes Júnior[5]; Filipe Martins Santos[1,3,6,7] Nayara Yoshie Sano[1]; João Vitor dos Santos Alves da Silva[8]; Victória Valente Califre de Mello[8]; Eliz Oliveira Franco[8]; Ana Cláudia Calchi[8,9]; Marcos Rogério André[8]; Carina Elisei De Oliveira[1,3]

[1]Parasitic Biology Laboratory, Department of Environmental Sciences and Agricultural Sustainability, Dom Bosco Catholic University, Campo Grande, MS, Brazil

[2]Trypanosomatid Biology Laboratory, Department of Parasitic Biology, Oswaldo Cruz Foundation, Rio de Janeiro, RJ, Brazil
[3]Department of Biotechnology, Dom Bosco Catholic University, Campo Grande, MS, Brazil
[4]Institute of Biosciences, Federal University of Mato Grosso do Sul, Campo Grande, MS, Brazil.
[5]Facultad de Ciencias Médicas, Universidad del Pacífico, Pedro Juan Caballero, Paraguay.
[6]Department of Local Development, Dom Bosco Catholic University, Campo Grande, MS, Brazil.
[7]Department of Microbiology, Immunology and Parasitology, Rio de Janeiro State University, Rio de Janeiro, RJ, Brazil.
[8]Vector-Borne Bioagents Laboratory, Department of Pathology, Reproduction and One Health, School of Agricultural and Veterinarian Sciences, São Paulo State University, Jaboticabal, SP, Brazil.
[9]Departament of Animal Biology, Biology Institute, State University of Campinas, Campinas, SP, Brazil.

Corresponding author e-mail: juliapistori1@gmail.com (Julia Gindri Bragato Pistori)
