# Supplementary material for: The role of capybaras (Hydrochoerus hydrochaeris) as hosts for hemoplasmas in an urban park of Brazilian Midwest
Source: Vet Res Commun. 2026 Nov 7;51(1):42. doi: 10.1007/s11259-026-11603-8 (PMC13744522; doi:10.1007/s11259-026-11603-8)
Supplement: Supplementary file 3 [file 11259_2026_11603_MOESM3_ESM.pdf]

Table S2 Hematological parameters of capybaras (Hydrochoerus hydrochaeris) sampled in Campo Grande, Mato Grosso do Sul, Brazil, positive and negative for hemoplasmas. Data are expressed as mean ± (SD) standard deviation.

Parameters	Infected (47)	Non-infected (10)	*p*-value	Distribution
RBC (10^{12}/L)	2.60 ± 0.51	2.64 ± 0.41	0.8058	> 0.05
Hb (g/L)	9.98 ± 1.66	10.10 ± 0.72	0.7448	< 0.05
Hct (L/L)	36.94 ± 3.78	38 ± 2.21	0.4602	< 0.05
MCV (f/L)	146.43 ± 29.31	147.64 ± 28.04	0.926	< 0.05
MCHC (g/L)	27.35 ± 5.77	26.60 ± 1.62	0.8502	< 0.05
MCH (g/L)	39.89 ± 11.35	39.25 ± 7.74	0.9164	< 0.05

The role of capybaras (*Hydrochoerus hydrochaeris*) as hosts for hemoplasmas in an urban park of Brazilian Midwest

Veterinary Research Communications

Julia Gindri Bragato Pistori[1]; Gabriel Carvalho de Macedo[1,2]; Heitor Miraglia Herrera[1,3]; William Oliveira de Assis[1]; Leonardo França do Nascimento[4]; Oscar Fernandes Júnior[5]; Filipe Martins Santos[1,3,6,7] Nayara Yoshie Sano[1]; João Vitor dos Santos Alves da Silva[8]; Victória Valente Califre de Mello[8]; Eliz Oliveira Franco[8]; Ana Cláudia Calchi[8,9]; Marcos Rogério André[8]; Carina Elisei De Oliveira[1,3]

[1]Parasitic Biology Laboratory, Department of Environmental Sciences and Agricultural Sustainability, Dom Bosco Catholic University, Campo Grande, MS, Brazil

[2]Trypanosomatid Biology Laboratory, Department of Parasitic Biology, Oswaldo Cruz Foundation, Rio de Janeiro, RJ, Brazil

[3]Department of Biotechnology, Dom Bosco Catholic University, Campo Grande, MS, Brazil

[4]Institute of Biosciences, Federal University of Mato Grosso do Sul, Campo Grande, MS, Brazil.

[5]Facultad de Ciencias Médicas, Universidad del Pacífico, Pedro Juan Caballero, Paraguay.

[6]Department of Local Development, Dom Bosco Catholic University, Campo Grande, MS, Brazil.

[7]Department of Microbiology, Immunology and Parasitology, Rio de Janeiro State University, Rio de Janeiro, RJ, Brazil.

[8]Vector-Borne Bioagents Laboratory, Department of Pathology, Reproduction and One Health, School of Agricultural and Veterinarian Sciences, São Paulo State University, Jaboticabal, SP, Brazil.

[9]Departament of Animal Biology, Biology Institute, State University of Campinas, Campinas, SP, Brazil.

Corresponding author e-mail: juliapistori1@gmail.com (Julia Gindri Bragato Pistori)
